# Application of Citizen Science Risk Communication Tools in a Vulnerable Urban Community

**DOI:** 10.3390/ijerph13010011

**Published:** 2015-12-22

**Authors:** Yuqin Jiao, Julie K. Bower, Wansoo Im, Nicholas Basta, John Obrycki, Mohammad Z. Al-Hamdan, Allison Wilder, Claire E. Bollinger, Tongwen Zhang, Luddie Sr. Hatten, Jerrie Hatten, Darryl B. Hood

**Affiliations:** 1 Division of Environmental Health Sciences, College of Public Health, The Ohio State University, Columbus, OH 43210, USA; jiao.70@buckeyemail.osu.edu (Y.J.); wilder.106@buckeyemail.osu.edu (A.W.); bollinger.69@buckeyemail.osu.edu (C.E.B.); zhang.5498@osu.edu (T.Z.); 2 Division of Epidemiology, College of Public Health, The Ohio State University, Columbus, OH 43210, USA; bower.185@osu.edu; 3 VERTICES, LLC 303 George Street Suite 406, New Brunswick, NJ 08901, USA; gis@vertices.com; 4 Environmental Science Graduate Program, School of Environment and Natural Resources, The Ohio State University, Columbus, OH 43210, USA; basta.4@osu.edu (N.B.); obrycki.2@buckeyemail.osu.edu (J.O.); 5 Universities Space Research Association at NASA Marshall Space Flight Center, Huntsville, AL 35805, USA; mohammad.alhamdan@nasa.gov; 6 Stambaugh-Elwood Citizens for the Environment, LLC Columbus, OH 43207, USA; luddiehattensr@gmail.com (L.S.H.); jerriehatten@gmail.com (J.H.)

**Keywords:** environmental justice, environmental justice index, toxics release inventory, environmental contaminants, health disparities, public health exposome, public participatory geographical information systems, MapplerX

## Abstract

A public participatory geographical information systems (PPGIS) demographic, environmental, socioeconomic, health status portal was developed for the Stambaugh-Elwood (SE) community in Columbus, OH. We hypothesized that soil at SE residences would have metal concentrations above natural background levels. Three aims were developed that allowed testing of this hypothesis. Aim 1 focused on establishing partnerships between academia, state agencies and communities to assist in the development of a community voice. Aim 2 was to design and conduct soil sampling for residents of the SE community. Aim 3 was to utilize our interactive, customized portal as a risk communication tool by allowing residents to educate themselves as to the potential risks from industrial sources in close proximity to their community. Multiple comparisons of means were used to determine differences in soil element concentration by sampling location at *p* < 0.05. The results demonstrated that eight metals (As, Cd, Cu, Pb, Mo, Se, Tl, Zn) occurred at statistically-significantly greater levels than natural background levels, but most were below risk-based residential soil screening levels. Results were conveyed to residents via an educational, risk-communication informational card. This study demonstrates that community-led coalitions in collaboration with academic teams and state agencies can effectively address environmental concerns.

## 1. Introduction

Within government, as well as the academic and scientific community, the focus has shifted towards more of an exposome framework approach when addressing current public health and environmental health issues. The public health exposome is a population-based, exposure science approach to health disparities research. This framework is an integrated model for examining exogenous and endogenous source-exposure-disease relationships across the life cycle and the influence of those relationships on health disparities at a population level [1]. 

The Healthy People 2020 campaign has identified five social determinants of health: economic stability; education; health and healthcare; neighborhood and built environment; and the social and community context. From an environmental health perspective, the neighborhood and built environment determinant has proven to contribute significantly toward assessing health outcomes that are believed to result from exposure to environmental contaminants. The assessment of environmental conditions is an important consideration in determining potential exposure of humans to hazardous or toxic substances that negatively impact our health [2].

The Kirwan Institute for the Study of Race and Ethnicity at Ohio State University similarly categorizes social determinants of health, but describes them from three perspectives: domains, levels and pathways. Akin to the Healthy People 2020 approach to evaluating health outcomes, the Kirwan Institute also recommends a broader focus that recognizes the relevance of specific areas, geographic scale and the actual mechanisms by which individuals or populations are affected by specific hazards or experiences. In order to address such important public health issues, recognition of structural barriers, as well as the identification of population susceptibility factors must occur when evaluating disparity issues [3]. 

### Environmental Health Disparities

There are numerous communities throughout the United States that bare a disproportionate burden of environmental hazards within close proximity to their living space [4]. This occurrence with respect to vulnerable communities is consistent with an “environmental injustice” theme.

To this end and historically, participatory GIS (P-GIS) has been proven to have robust potential as a tool for analyzing and mapping indicators of “poverty”, “exclusion” or “discrimination” within rural and urban communities. The disenfranchised populations of our society can be mapped as distinct spatial sites or as zones of disparities. Applications from public participatory geographical information systems (PPGIS) practice and research include: mapping “environmental racism”, *i.e.*, the spatial correlation between environmental degradation and the distribution of ethnic or socio-economic groups in urban areas (e.g., Stuart [5]; Kellogg [6]); social equity mapping,* i.e.*, the identification of socio-economic groups that are relatively disadvantaged by ethnicity, employment status, caste, economic class, age, language location or by gender. As reviewed by Hall [7] and Kwan [8], PPGIS utilization has been employed for analyzing differential mobility and people’s access to services according to social categories. Burke and Sawicki [9] and Poole [10] noted in seminal reports that a significant component of P-GIS empowers marginalized groups. Additionally, making such geo-information available serves to promote transparency in decision-making processes [11]. 

The Environmental Protection Agency (EPA) defines environmental justice (EJ) as, “the fair treatment and meaningful involvement of all people regardless of race, color, national origin, or income with respect to the development, implementation, and enforcement of environmental laws, regulations, and policies” [12]. Often, communities that are disproportionally exposed to an excess of environmental hazards may also be lacking in the other social determinants of health: economic stability, education, healthcare and the social context [13,14]. Low-income communities and specific minority groups are more frequently burdened by the cumulative effects of additional stressors and more frequent exposure pathways [15]. 

Although environmental justice is something to strive for in all communities, the visibility of the environmental justice movement did not begin until the late 1960s following the American Civil Rights movement. There were several landmark cases in the 1970s and 1980s that were indicative of environmental injustice in communities with certain demographics. For example, in 1979, Bean v. Southwestern Waste Management Inc. was one of the first lawsuits to challenge waste facility siting within a community. Three years later, the placement of a polychlorinated biphenyl landfill placed in Warren County, North Carolina, led to a large number of community member protests. This particular incident steered research studies that assessed predictive factors, such as race, socioeconomic status, land values and the relationship with facility siting and proximity to emissions from industrial facilities. In 1990, Robert Bullard, coined the “father of environmental justice”, fused the concepts of social determinants, environmental movements and community-led resistance strategies in his book Dumping in Dixie: Race, Class and Environmental Quality. With the goals of environmental equity, Bullard planned the 1991 National People of Color Environmental Leadership Summit that highlighted a more comprehensive approach in viewing environmental health issues. The summit’s focus of “public health, worker safety, land use, transportation, housing, resource allocation, and community empowerment” (p.556) ultimately led to the core principles of environmental justice [16]. 

Despite the growth and visibility of environmental justice movements, millions of people today still experience the burden of excessive hazards within their communities. The EPA has identified seven factors that may contribute to environmental factors in vulnerable communities [17]: (in no particular order) (1) chronic psychosocial stress; (2) unique exposure; (3) multiple and cumulative environmental burdens; (4) physical infrastructure; (5) diminished capacity to participate in decision making; (6) vulnerability/susceptibility; and (7) proximity to sources of environmental hazards.

The Stambaugh-Elwood community is one community of seven within the Southern Gateway in Columbus, Ohio, that exhibits many of these characteristics that may contribute to the disparate health outcomes observed in this community. Our preliminary assessment of the heavy metal content of soils in this urban area that is located in close proximity to industrial complexes represents the first step in determining a hazard index for this area. Although heavy metals may be present naturally in soil in trace amounts, there may be potential health risks to humans if levels are in excess of Franklin County, OH, background levels. Anthropogenic and industrial processes have been sown to increase the potential for adverse exposure to human populations [18,19]. Cumulative and chronic exposures may be contributing factors to a variety of health ailments. Exposure to heavy metals can negatively impact normal functioning of the reproductive and central nervous systems, result in fetal and/or infant death, low birth rates and potential developmental issues [18,19,20,21]. In addition, arsenic, cadmium, chromium and lead are classified as known or probable human carcinogens by the International Agency for Research on Cancer [22]. 

A prior study assessed heavy metal concentrations in a community located approximately 8–10 miles from Stambaugh-Elwood [23]. Heavy metal concentrations (As, Cd, Cr, Pb and Zn) were compared to the USEPA regional soil screening levels. The study identified Pb and Zn as metals that exceeded Franklin County background concentrations. Similarly to Stambaugh-Elwood, Weinland Park is considered to be a post-industrial low-income urban neighborhood. Fifty percent of Weinland park residents identify as African American. The average annual income of the neighborhood was $17,000 in 2009. About 36% of residents are not employed, and for those that are employed, 18% of residents have full-time employment. The average for the City of Columbus is $41,370 [23,24]. 

The linkage between environmental domains and disparate health outcomes is gaining appreciation for its multi-dimensionality and defines the recent introduction of our public health exposome framework [1,25]. According to several studies, socioeconomic status may also be a contributing factor to adverse health outcomes [26,27]. Residents of Franklin County, OH, have an average household income of $67,000 annually, and the average Columbus residential home earns approximately $55,000. Southern Gateway residents have an average annual household income of approximately $33,000, which is significantly less than Franklin County and Columbus city averages. In the Southern Gateway community, the poverty rate is 43%, about two times higher than the Columbus city average (~22%) [28]. 

Like Weinland Park, the Stambaugh-Elwood community characteristics are also indicative of a post-industrial low-come urban neighborhood. In the 1970s, many of the factories and businesses that had thrived during the Industrial Era began to close. The long-term economic effects can be seen within the community as the economy has shifted. Residents have expressed concerns about air quality due to nearby Columbus Castings, one of the metal scrap plants that remain on the South Side. The Stambaugh-Elwood community consists of 46% residents that identify as African American, whereas the overall percentage of the African American population for Columbus, OH, is 28%. This community also has a significantly higher elderly population of 15% of the resident population. Annual income rates show that African Americans have an adjusted mean income of $14,273, whereas non-Hispanic white residents have more than double the income, with an adjusted mean income of $30,451. Overall, 72% percent of residents live below the poverty line. According to comments made by the Southern Gateway Collaborative committee, Stambaugh-Elwood (SE) is isolated from the rest of the Southern Gateway due to industrial use, and the community has been “neglected in terms of infrastructure improvements” [29]. Many residents live near industrial facilities that contain hazardous substances. Adverse health outcomes are much more prevalent in SE than other parts of the city [28]. Recent studies substantiate a growing concern related to potentially linking the spatiotemporal relationships of social vulnerability and exposure to environmental contaminants [30,31]. In a later study, Krieger and colleagues in an elegant study demonstrated that neighborhoods with higher concentrations of poverty and populations of color have higher exposure to traffic-related pollutants [31]. In fact, the study improves our understanding of the social determination of these patterns by analyzing exposure to one spatially-patterned traffic-related air pollutant, black carbon, in connection to the Index of Concentration at the Extremes (ICE) [32]. The focus on black carbon as an exposure with notable public health significance was informed by a plethora of evidence indicating that it is “causally involved in all-cause, lung cancer, and cardiovascular mortality, morbidity, and likely adverse birth outcomes and nervous system effects” [33]. Within the United States, the greatest single contributor to black carbon ambient levels is transportation (2010 estimate: 216 out of 321 Gg emitted) [33], rendering black carbon an important indicator of ambient traffic-related fine particulate matter, especially for urban air pollution [33,34]. This study represents the first phase in deploying our public health exposome framework to analyze potential exposure to spatially-patterned soil contaminant levels in connection to the ICE.

The ICE simultaneously measures the concentration of privilege and deprivation and can be computed at multiple levels and scales. The results of the Krieger* et al.* [31] study suggest that extreme concentrations of socioeconomic resources and racial/ethnic privilege, structured by social class and race relations, as manifested at the census tract level, are inversely associated with individuals’ residential exposure to black carbon, an important airborne pollutant, even after controlling for individual and household social characteristics.

Vulnerable communities often bear the burden of environmental injustice within their communities, due to political, economic and social factors [35,36,37]. Various population-based studies have shown the associations of environmental disparities being linked to socioeconomic status and race [38,39,40,41]. Studies have shown that communities exhibit various degrees of health outcomes that have previously been linked to environmental contaminants [29,42,43]. For example, preterm birth and infant mortality are adverse health outcomes that have links to environmental exposures. As reported in Ferguson* et al.*, 2013 [44], there are suggestive associations of environmental contamination/exposures that may impact preterm birth outcomes. The heightened concern from environmental contamination and its effect on the fetus is reasonable because of the potential for long-term exposure and the ability of certain contaminants to cross the placental barrier [44].

**Figure 1:**
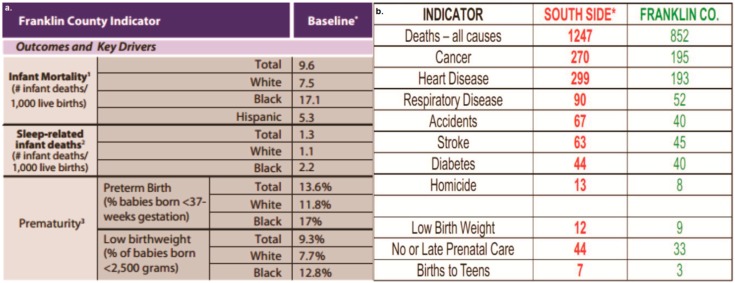
(**a**) Franklin County (Franklin CO.) infant birth outcomes [36]. (**b**) Comparison between South Side health statistics and Franklin County (birth and prenatal care numbers noted in percentages) [3,45,46,47,48].

SE residents live within close proximity to facilities that harbor harmful environmental contaminants. Franklin County, Ohio, has one of the highest infant mortality rates in the nation. Although the Franklin County infant mortality rate is already extraordinarily high in comparison to the rest of the nation, there is also a noticeable disparity between the SE neighborhood within the Southern Gateway and the rest of the county. Franklin County has a 9% low birth weight rate, whereas the South Side has a 12% low birth weight rate (Figure 1).

## 2. Materials and Methods 

### 2.1. Community Mapping Decision Support Tools 

**Figure 2:**
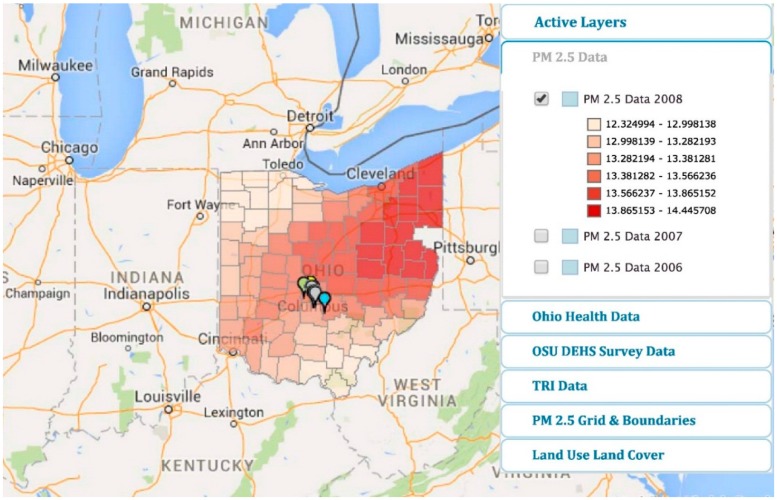
Spatiotemporal presentation of PM_2.5_ data from the 88 counties in Ohio from the NASA MODIS satellite available at MapplerX [49]. TRI, Toxic Release Inventory. OSU, The Ohio State University; DEHS, Division of Environmental Health Sciences.

Subsequent to the development and customization of a PPGIS portal, MapplerX [49] (Figure 2), by the Division of Environmental Health Sciences, College of Public Health in April 2014, the Environmental Protection Agency launched EJSCREEN in June 2015. Like MapplerX, EJSCREEN is an interactive mapping tool that enables residents of Southern Gateway communities in Columbus, OH, to access public and private databases that may assist in identifying communities that may have environmental concerns. The EPA tool EJSCREEN combines twelve environmental indicators and six demographic indicators that combine to create twelve environmental justice indices. The data include pollution exposure estimates and are able to show population proximity to industrial or facility locations. This comprehensive tool enables users to access a variety of data; however, it is important to note that EJSCREEN is not to be used as a risk assessment tool. Rather, it can highlight areas of interest that may be burdened by environmental disturbances and health disparities. Due to the fact that the EJSCREEN maps can provide data at the local level, they serve to provide the much-needed attention to vulnerable communities that may be in need of further scientific review, analysis or policy changes [50,51].

**Figure 3:**
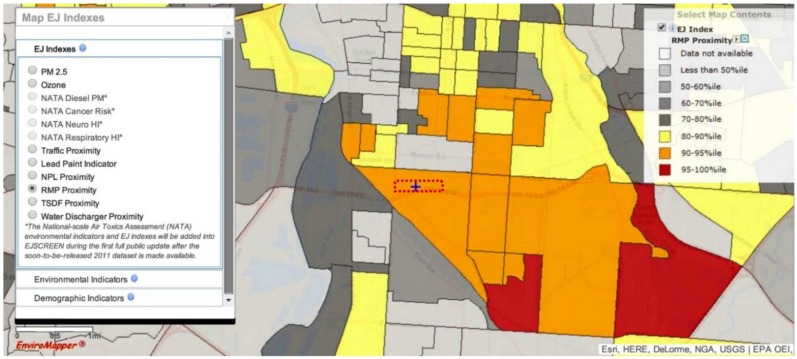
EPA-EJSCREEN portal. Stambaugh-Elwood (SE) population (*n* = 150) is in the 90–93 percentile for the proximity to a facility with a Risk Management Plan (RMP). The red rectangle denotes the SE neighborhood. EJ: environmental justice; The National-scale Air Toxics Assessment (NATA); Proximity National Priority List Sites (NPL); Proximity to Treatment Storage and Disposal Facilities (TSDF) [51].

The SE community of Columbus, Ohio (*n* = 150) is located within the zip code 43207. Within EJSCREEN, there is approximately 90%–95% of this area within close proximity to a facility requiring a Risk Management Plant (RMP) by the USEPA (Figure 3). Under the Clean Air Act, facilities that use extremely hazardous substances are required to have an RMP on file. For community members that live within the vicinity of these facilities, it is possible that hazardous outputs might be contributing factors to disparate health outcomes within the region [52].

The present preliminary study sought to identify metalloid levels from an urban soil matrix in a community that is located in close proximity to industry, thereby providing baseline residential soil level data for residents and other stakeholders. An important aspect of evaluating relationships between the built environment and health outcomes is geographical information about structural points of interest within a neighborhood. A mapping database, such as geographic information systems (GIS), can provide insight for a variety of potential contributing factors, such as environmental management, social economic status and proximity to potential hazards. PPGIS is a community engagement form of GIS that can also assist with public health advocacy [53,54]. 

Information provided on the portal developed for the SE community is shown in Table 1 and includes: (1) Ohio health data from the Ohio Department of Health database; (2) sub-county-level gridded PM_2.5_ data from the Marshall Space Flight Center database in Huntsville, AL [55]; (3) USEPA (Unites States Environmental Protection Agency) Toxic Release Inventory (TRI) data and EPA air data from approximately twelve industrial sites within the boundaries of Southern Gateway communities comprising zip codes 43206 and 43207; (4) census track and zip code data; and (5) land use and cover. An added feature for stakeholders is the ability to permit the uploading of individual spatial data specific to an individual’s place of residence. 

**Table 1:** Examples of mineable sources of environmental data (bold) that are presently included in the customized public participatory geographical information systems (PPGIS) portal for Stambaugh-Elwood.

Dataset	Type of Data	Partner Providing Access (or Publically Available)
Medicaid; Ohio Medicaid Assessment Survey	Claims data; insurance, access to care, health behaviors and experiences of care	Nationwide Children’s Hospital (NCH), Government Resource Center (GRC), Medicare & Medicaid Services
Pregnancy Risk Assessment Monitoring System (PRAMS)	Pre- and peri-natal risk and exposure data	Publicly available
USDA Food Environment Atlas	Access to affordable food, food security	Publicly available
U.S. Census	Socioeconomic data	Publicly available
NASA PM_2.5_	Air quality data for all 88 Ohio counties	Marshall Space Flight Center
Birth and death certificates	Detailed birth outcomes data; can be linked	Ohio Department of Health
Tobacco retail outlet data	Exposure to tobacco advertising	Nielsen, County Auditors
Expression Genetics in Drug Therapy (XGEN)	Genome-wide data on disease phenotypes	OSU Center for Pharmacogenomics
Phenotypes and eXposures (PhenX)	Phenotype database	NIH
OH Longitudinal Data Archive	P-20 education data; housing data	OSU Center Human Resource Research
Adolescent Health & Development in Context	Socio-spatial study of child Adolescent health in Franklin Co	OSU Department Sociology
Epic Systems Corporation (EPIC)	Electronic health records	Wexner Medical Center, NCH
Toxics Release Inventory	Smokestack emissions into environment	Publicly available

### 2.2. Consent for Soil Sampling

The consent process for soil sampling at SE residences for this study was done in collaboration with the monthly meetings of the South Side Health Advisory Committee. The South Side Health Advisory Council (SSHAC) is a community health committee sanctioned by Columbus Public Health that seeks to bring the health concerns of a community to the forefront. This specific committee serves residents of the zip codes 43205, 43206 and 43207. Every third Thursday of the month, the Southside Health Advisory Councils hosts community engagement meetings at the Church for All People, located at 946 Parsons Ave, Columbus, OH 43206. Residents of the Southern Gateway community attended these meetings and were provided with project background information from The Ohio State University researchers. During these meetings, residents were given the opportunity to decide if they would like to consent for soil sampling at their residence. Residents were informed of the benefits of the research, potential risks and protection against risks. Although this project was determined to not include human subjects, all residents that elected to take part in the research did sign a consent form: Institutional Review Board, Protocol #2014B0445 (exempt). Consented participants were made aware that they: (1) could leave the study any time; (2) could decide to stop participating in the study and, if so, that there will be no penalty to them; and (3) their decisions would not affect their future relationship with the College of Public Health at The Ohio State University [56]. Forty-two residences consented to participate in the study. There are approximately 150 people in the Stambaugh-Elwood community. From month to month over a two-year period, 35–50 residents attended the monthly meeting of the South Side Health Advisory Committee, which is a sub-committee of Columbus Public Health. The demographic breakdown of the Stambaugh-Elwood community is (1) African American and (2) gender balanced, with the majority of the population over the age of 50. From the individuals that consented to participate in the soil sampling research, future appointments were made for a research team member to obtain soil samples at the residence. 

### 2.3. Soil Sampling Strategy 

**Figure 4:**
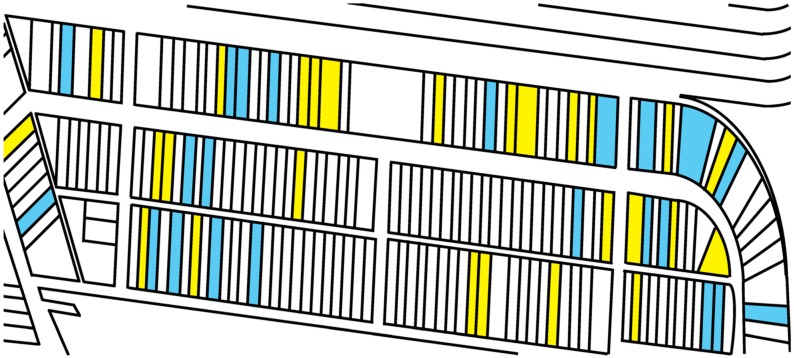
A random generator algorithm selected SE residences for soil sampling (blue residences). See the text for details.

Twenty-one resident locations within the SE neighborhood were selected from consenting residences by using a random number generator (Figure 4). The selected residences were split into east and west halves of the neighborhood. Ten–eleven residences from each half were selected for a total of 21 residential locations. All soil sampling occurred on 11 April 2015. During sampling, the researchers used their judgment as to shifting the sampling strategy to ensure the better overall balance of sampling coverage in the neighborhood. For example, the number generator selected two residences immediately adjacent to one another. A substitution occurred to sample from a residence farther away.

**Figure 5:**
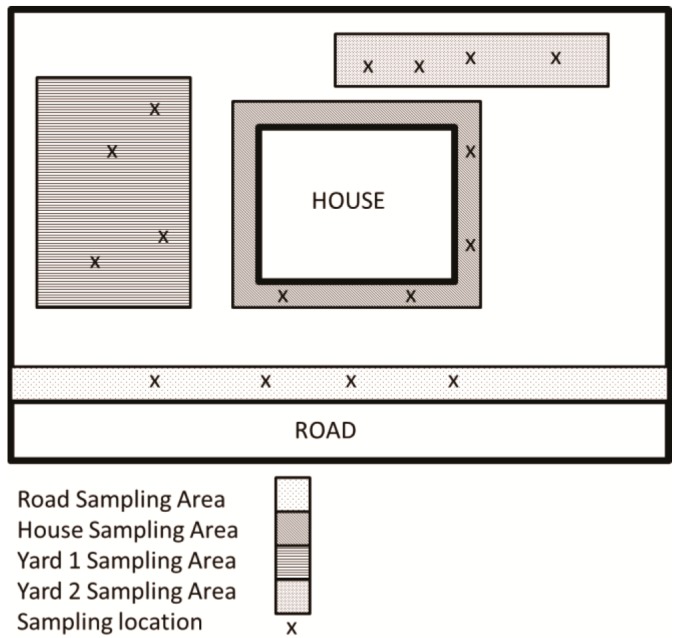
Schematic of the soil sampling strategy for selected residences in the Stambaugh-Elwood community.

As referenced in Figure 5 (below), at each location, four types of soil samples were collected: house, road, Yard 1 and Yard 2. For the house samples: four subsamples were collected along the foundation and placed into the house sample bag. For the road samples, four subsamples were collected from the yard area along the road and placed in the road sample bag. For the Yard 1 sample, four subsamples were collected from the general yard area and placed in the Yard 1 sample bag. For the Yard 2 sample, four subsamples were collected from a different part of the yard and placed in the Yard 2 sample bag. Soils were collected using bulb planters, to a depth of 2 inches. There were 84 total samples collected from all of the SE residences. Acquisition of soil samples was performed for total metal content using EPA Method 3051A [57,58]. In totality, sampling resulted in 84 soil (4 × 21) samples. Each sample consisted of four subsamples. For example, walking along the foundation and placing the bulb planter into the soil at four different locations represented the house foundation sample. All of these four subsamples were placed and included in the house-sampling bag. Samples were transported to the laboratory of Nicholas Basta, Ph.D., at Ohio State University and allowed to dry under a fume hood for approximately 2 weeks. Samples were subsequently sieved to 2 mm, and a subsample was crushed using a mortar and pestle for a total acid digestion following EPA Method 3051A. Soil digests were subsequently analyzed for metals using an Agilent 700 ICP-OES (Agilent Technologies, Santa Clara, CA, USA).

### 2.4. Statistical Analysis of Data 

The total mean metal content of soils was compared to upper prediction limit values, either as reported by The Ohio Environmental Protection Agency (OEPA) (2013) or calculated from the 95th percentile from U.S. Geological Survey (USGS) (2004, 2013). Soils were considered elevated if the element concentration + 2 standard deviations (SD) were above the 95th percentile natural background concentration reported by USGS (2004, 2013) or OEPA (2013). The minimum, maximum and percentage elevation effect size was calculated for soils with elevated of elements. Multiple comparisons of means were used to determine differences in soil element concentration by sampling location (*i.e.*, house* vs.* road* vs.* yard) at *p* < 0.05. This was accomplished by analysis of variance (ANOVA) and subsequent analysis using Tukey’s honest significant difference (HSD) test at *p* < 0.05. 

## 3. Results and Discussion

The citizen science GIS portal that was developed for Southern Gateway communities can be viewed in real time at MapplerX [49]. Presently, our tool allows for mapping, visualization and communicating spatial information from large public and private environmental datasets that are relevant to the health status of Southern Gateway community residents. This methodology is a promising approach for engaging community partners in educational risk communication and/or hazard identification assessments by providing an on-line, interactive mapping functionality. As can be seen in the active layer legend in the portal, this functionality allows stakeholders to map data from: (1) health and/or environmental surveys, (2) black/white infant mortality hot spots [59]; (3) Toxics Release Inventory (TRI) data [27]; (4) NASA (National Aeronautic Space Administration) MODIS satellite PM_2.5_ sub-county level gridded data; (5) zip codes; (6) Ohio census tracks; and (7) land use/land cover data. The methodology also allows stakeholders to map disparities, population and community characteristics and risk factors that are gathered during consolidation of a community principle [16]. In the case of the SE community, our citizen science approach to mapping this community within the context of the larger community presented an opportunity to propose that this population was vulnerable simply based on proximity to potential contaminant emissions from neighboring industry. This supposition was validated with the release of the USEPA EJSCREEN tool. Upon searching the zip code 43207 in EJSCREEN, this census track area scored over 80% in the overall (1) Environmental Justice Index, (2) demographic and (3) environmental profiles to be consistent with the characteristics of an environmental justice community.

### 3.1. Data Mining for Historical Industrial Emissions in Close Proximity to SE 

**Figure 6:**
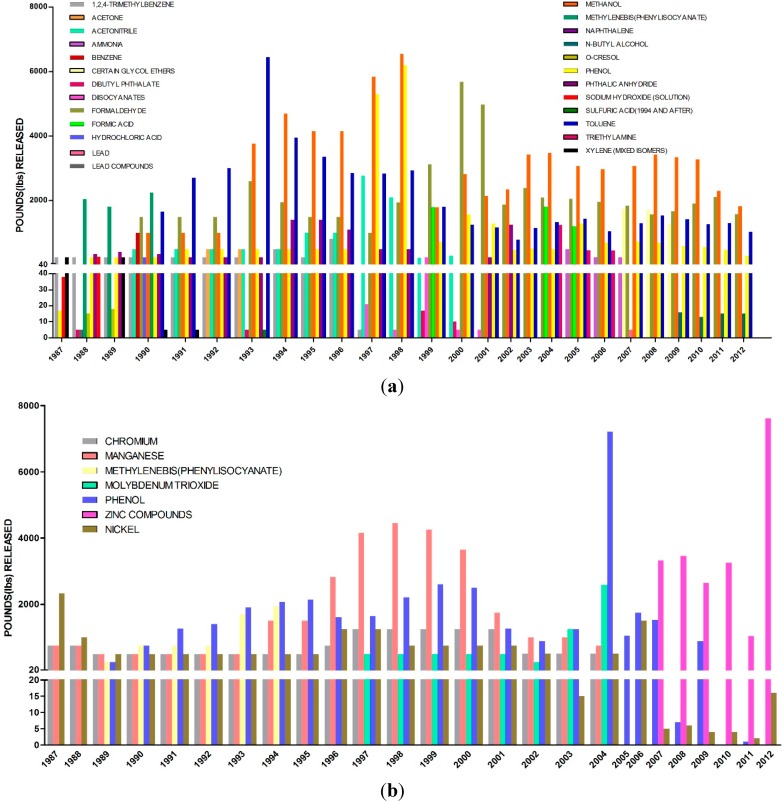
USEPA Toxic Release Inventory data (1987–2012) of on-site releases for (**a**) Company A and (**b**) Company B located in close proximity to the Stambaugh-Elwood Community.

The two graphs below are examples of how our PPGIS portal can assist vulnerable populations in communities located in close proximity to industrial facilities in educating themselves as it relates to historical contaminant emissions from neighboring industrial facilities. Figure 6a represents the total contaminant air releases from an industrial company in zip code 43207 in close proximity to the SE community for the period 1987–2012. Shown are the data plotted from the TRI database for this 26-year period. 

Figure 6b represents the total contaminant emissions from a second industrial facility (Company B) in zip code 43207 for the same 25-year period from 1987 to 2012. From 1987 to 2002, releases of chromium, manganese and manganese containing compounds, molybdenum trioxide, nickel and zinc comprise the majority of releases for the eight-year period from 1996 to 2004, totaling more than a combined 30,000 total pounds released into the environment. Naphthalene (a polycyclic aromatic hydrocarbon whose neurodevelopmental and reproductive toxicity has been thoroughly investigated over the past 20 years) [60,61,62,63,64,65,66,67,68] releases into the environment also far exceed 20,000 pounds per year for this eight-year period. 

### 3.2. Soil Sampling Results in the SE Community

**Figure 7:**
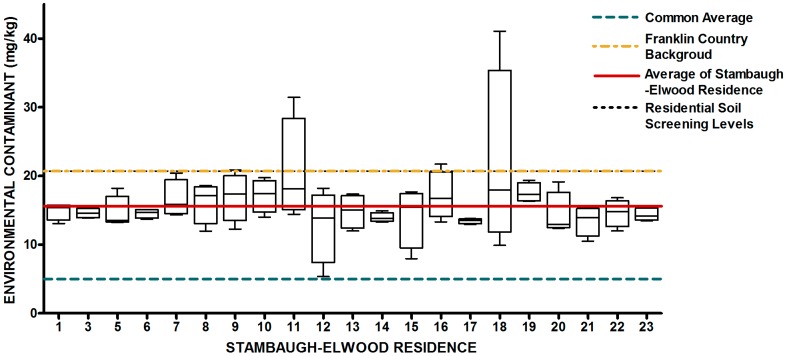
Residential urban soil level of As in the Stambaugh-Elwood community versus Franklin County (background), common averages and residential screening levels (RSLs) (mg/kg soil). Soil samples were taken from 21 of 46 residences that consented for the study in compliance with OSU IRB Protocol 2014B0445-exempt. Each sample consisted of four subsamples. Analysis proceeded following EPA Method 3051A, and digests were subsequently analyzed for metals using an Agilent 700 ICP-OES.

**Figure 8:**
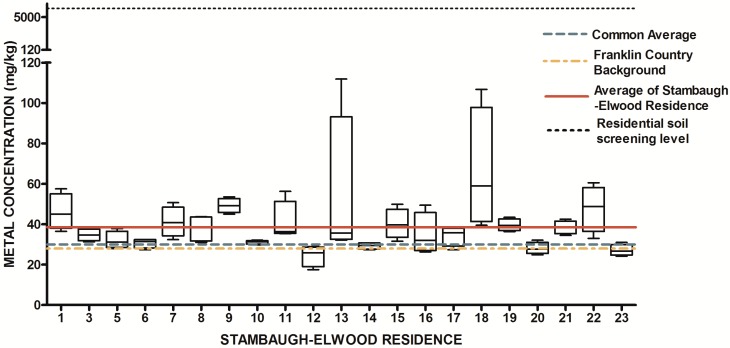
Residential urban soil level of Cu in the Stambaugh-Elwood community versus Franklin County (background), common averages and residential screening levels (RSLs) (mg/kg soil). Soil samples were taken from 21 of 46 residences that consented for the study in compliance with OSU IRB Protocol 2014B0445-exempt. Each sample consisted of four subsamples. Analysis proceeded following EPA Method 3051A, and digests were subsequently analyzed for metals using an Agilent 700 ICP-OES.

**Figure 9:**
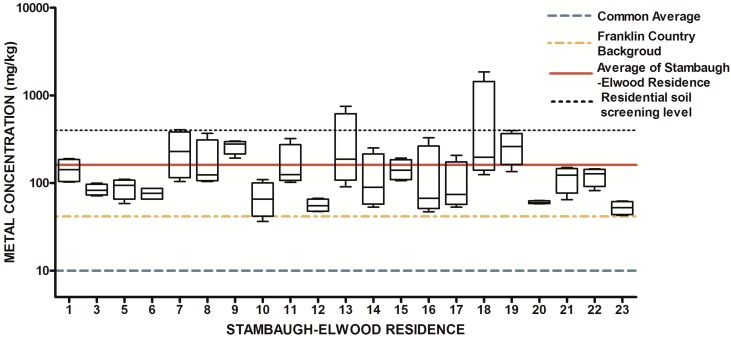
Residential urban soil level of Pb in the Stambaugh-Elwood community versus Franklin County (background), common averages and residential screening levels (RSLs) (mg/kg soil). Soil samples were taken from 21 of 46 residences that consented for the study in compliance with OSU IRB Protocol 2014B0445-exempt. Each sample consisted of four subsamples. Analysis proceeded following EPA Method 3051A, and digests were subsequently analyzed for metals using an Agilent 700 ICP-OES.

**Figure 10:**
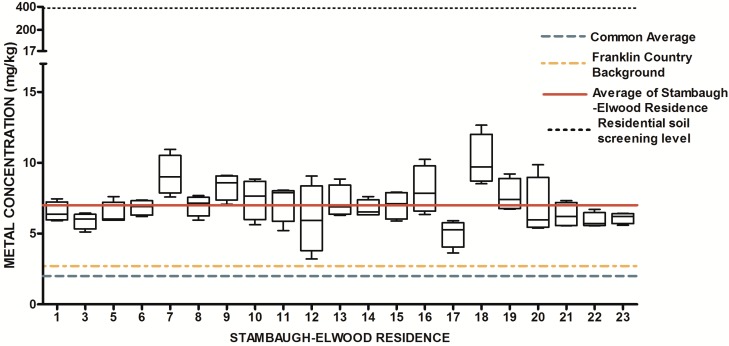
Residential urban soil level of Mo in the Stambaugh-Elwood community versus Franklin County (background), common averages and residential screening levels (RSLs) (mg/kg soil). Soil samples were taken from 21 of 46 residences that consented for the study in compliance with OSU IRB Protocol 2014B0445-exempt. Each sample consisted of four subsamples. Analysis proceeded following EPA Method 3051A, and digests were subsequently analyzed for metals using an Agilent 700 ICP-OES.

**Figure 11:**
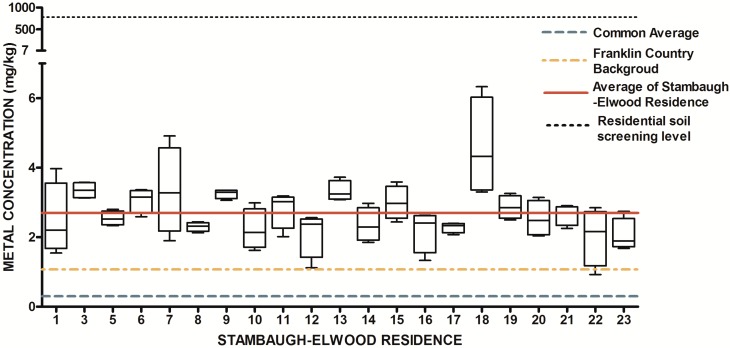
Residential urban soil level of Se in the Stambaugh-Elwood community versus Franklin County (background), common averages and residential screening levels (RSLs) (mg/kg soil). Soil samples were taken from 21 of 46 residences that consented for the study in compliance with OSU IRB Protocol 2014B0445-exempt. Each sample consisted of four subsamples. Analysis proceeded following EPA Method 3051A, and digests were subsequently analyzed for metals using an Agilent 700 ICP-OES.

**Figure 12:**
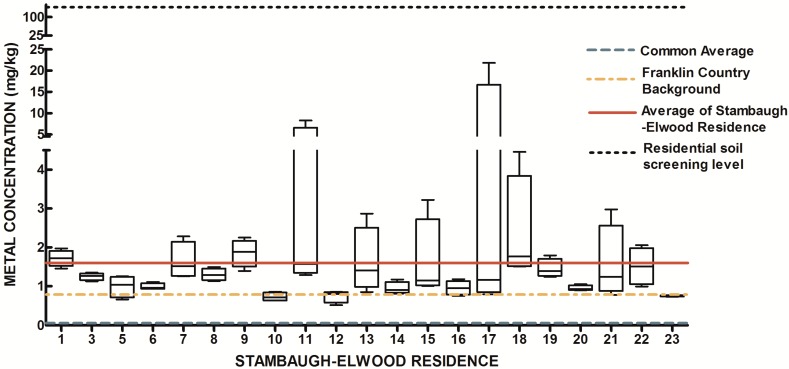
Residential urban soil level of Cd in the Stambaugh-Elwood community versus Franklin County (background), common averages and residential screening levels (RSLs) (mg/kg soil). Soil samples were taken from 21 of 46 residences that consented for the study in compliance with OSU IRB Protocol 2014B0445-exempt. Each sample consisted of four subsamples. Analysis proceeded following EPA Method 3051A, and digests were subsequently analyzed for metals using an Agilent 700 ICP-OES.

**Figure 13:**
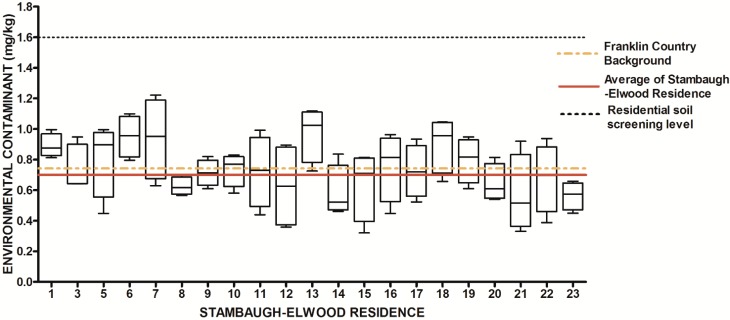
Residential urban soil level of Ti in the Stambaugh-Elwood community versus Franklin County (background), common averages and residential screening levels (RSLs) (mg/kg soil). Soil samples were taken from 21 of 46 residences that consented for the study in compliance with OSU IRB Protocol 2014B0445-exempt. Each sample consisted of four subsamples. Analysis proceeded following EPA Method 3051A, and digests were subsequently analyzed for metals using an Agilent 700 ICP-OES.

**Figure 14:**
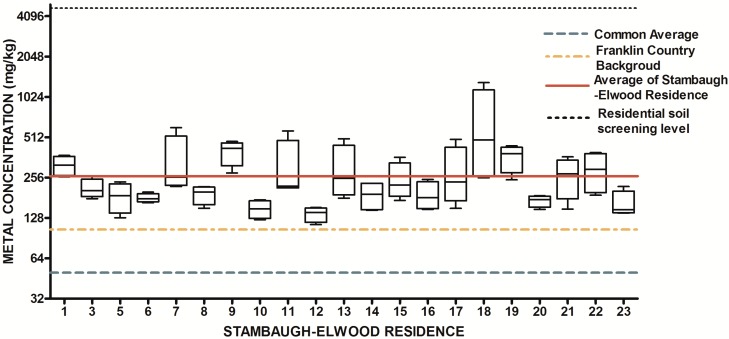
Residential urban soil level of Zn in the Stambaugh-Elwood community* versus* Franklin County (background), common averages and residential screening levels (RSLs) (mg/kg soil). Soil samples were taken from 21 of 46 residences that consented for the study in compliance with OSU IRB Protocol 2014B0445-exempt. Each sample consisted of four subsamples. Analysis proceeded following EPA Method 3051A, and digests were subsequently analyzed for metals using an Agilent 700 ICP-OES.

The design of our study allowed us to ask two fundamental research questions with regard to urban soils. First, is there a systematic elevation of soil metals and metalloids in the study area relative to expected concentrations from previously released findings (Lindsay, 1979 [69]; OEPA, 2013; USGS, 2004; USGS 2013) Since metals have natural abundance in all soils, it is important to compare background levels with measured soil levels to determine if contamination as evidenced by elevated levels of metals is present. The results from the 84 soil samples were compared to published values for soil metals and metalloids in Ohio and within Franklin County, OH (Table 1) (Figure 7, Figure 8, Figure 9, Figure 10, Figure 11, Figure 12, Figure 13 and Figure 14). The published values for soil metals in Ohio and Franklin County, OH, were not collected from areas impacted by human activities,* i.e.*, urban residential areas. The published values represent natural uncontaminated soil background levels of metals in soil. It is likely that human activities in urban areas will elevate metal levels. In the present study, seven metals and one non-metal (Cd, Cu, Pb, Mo, P, Se, Tl, Zn) occurred at levels greater than natural background levels (Table 2). 

**Table 2:** Comparison between metal concentrations in Stambaugh-Elwood soil* versus* published values.

Element	Mean ± 2 SE mg/kg	Upper Prediction Limits, mg/kg	Percent of Samples Exceeding Background 95th Percentile
	**All Samples (*n* = 84)**	**Published Values ^†^**	**Percent ^‡^**
Aluminum	12,670 ± 532	50,540 ^3^	
Arsenic	16 ± 1.0	20.7 ^2^	
Barium	143 ± 10	443 ^3^	
Beryllium	<0.1	1.5 ^3^	
Cadmium	1.6 ± 0.6	0.789 ^2^	86%
Chromium	30 ± 2	41.8 ^3^	
Cobalt	10 ± 0.4	12.2 ^3^	
Copper	38 ± 4	20.7 ^3^	49%
Iron	22,070 ± 580	39,098 ^1^	
Lead	160 ± 48	41.5 ^2^	99%
Magnesium	7120 ± 640	9777 ^1^	
Manganese	599 ± 28	765 ^1^	
Molybdenum	7 ± 0.4	2.7 ^3^	50%
Nickel	29 ± 2	36.4 ^2^	
Phosphorus	1050 ± 188	560 ^1^	99%
Potassium	4191 ± 196	16,657 ^3^	
Selenium	2.7 ± 0.2	1.07 ^2^	99%
Thallium	0.7 ± 0.04	0.743 ^2^	52%
Vanadium	35 ± 2	66 ^3^	
Zinc	262 ± 36	105 ^1^	94%

^†^ References: ^1^ USGS, 2004; ^2^ OEPA, 2013; ^3^ USGS, 2013. Upper prediction limit values either as reported by OEPA (2013) or calculated from the 95th percentile from USGS (2004, 2013). ^‡^ Calculated as (minimum effect size/UPL or 95th percentile) **times** 100 and (maximum effect size/UPL or 95th percentile) **times** 100 [70,71,72].

The second question that our design allows us to answer is: are there differences in the concentrations of soil metals and metalloids between the sampling locations (house, yard and road). The results from the 84 soil samples were compared to one another based on sampling location (Table 3). This resulted in 21 house samples, 21 road samples and 42 yard samples (21 for Yard 1 and 21 for Yard 2 combined). Most of the elements had consistent concentrations amongst the three sample locations. Zinc was elevated in the house soil samples relative to the other soil sampling locations. Cadmium and lead in house samples tended to have increased soil concentration compared to the other locations, but the increased concentration was not significant at *p* < 0.05. One element elevated in the road samples was magnesium. The statistically-significantly designations within an element can be seen in Table 3 and are represented by *p* < 0.05.

**Table 3:** Elemental metal concentrations in Stambaugh-Elwood soil sampling locations.

Element	Sample Location Mean (mg/kg) ± SE ^‡^			
	**House**	**Road**	**Yard 1**	**Yard 2**
Aluminum	12513 ± 438 ^a,‡^	11400 ± 669 ^b^	13221± 523 ^a,b^	13530 ± 356 ^a,b^
Arsenic	17.2 ± 1.3 ^a^	13.8 ± 1.1 ^b^	15.7 ± 0.5 ^a,b^	15.9 ± 0.5 ^a,b^
Barium	160 ± 16.6 ^a^	136 ± 6.3 ^a^	139 ± 4.4 ^a^	138 ± 5.2 ^a^
Beryllium	<0.1 ^a^	<0.1 ^a^	<0.1 ^a^	<0.1 ^a^
Cadmium	2.9 ± 1.0 ^a^	1.1 ± 0.1 ^a^	1.2 ± 0.1 ^a^	1.3 ± 0.1 ^a^
Chromium	31.9 ± 3.3 ^a^	29.9 ± 2.5 ^a^	29.2 ± 1.6 ^a^	27.6 ± 0.8 ^a^
Cobalt	10.3 ± 0.3 ^a^	8.4 ± 0.4 ^b^	9.8 ± 0.3 ^a^	9.8 ± 0.3 ^a^
Copper	44.8 ± 4.2 ^a^	36.7 ± 3.8 ^a^	36.4 ± 1.6 ^a^	36.0 ± 1.9 ^a^
Iron	23,100 ± 504 ^a^	20,295 ± 638 ^b^	22,406 ± 540 ^a^	22,478 ± 473 ^a^
Lead	251 ± 88 ^a^	116 ± 15 ^a^	145 ± 21 ^a^	130 ± 19 ^a^
Magnesium	7672 ± 526 ^b^	9692 ± 648 ^a^	5493 ± 406 ^c^	5625 ± 494 ^c^
Manganese	629 ± 23.3 ^a^	549 ± 24.5 ^a^	619 ± 32 ^a^	599 ± 30 ^a^
Molybdenum	7.8 ± 0.4 ^a^	6.3 ± 0.3 ^b^	7.0 ± 0.3 ^a,b^	7.1 ± 0.2 ^a,b^
Nickel	30.4 ± 0.8 ^a^	28.6 ± 2.8 ^a^	28.2 ± 0.7 ^a^	28.4 ± 0.7 ^a^
Phosphorus	951 ± 92.0 ^a^	1216 ± 365.3 ^a^	1018 ± 40 ^a^	1014 ± 40 ^a^
Potassium	4088 ± 166 ^a^	4141 ± 237 ^a^	4161 ± 191 ^a^	4372 ± 191 ^a^
Selenium	2.8 ± 0.2 ^a^	2.6 ± 0.2 ^a^	2.9 ± 0.2 ^a^	2.7 ± 0.1 ^a^
Thallium	0.7 ± 0.0 ^a^	0.7 ± 0.04 ^a^	0.8 ± 0.1 ^a^	0.8 ± 0.0 ^a^
Vanadium	34.6 ± 1.0 ^a^	32.2 ± 1.5^a^	36.0 ± 1.2 ^a^	36.4 ± 0.8 ^a^
Zinc	365 ± 57.0 ^a^	228 ± 27.7^b^	230.4 ± 17.7 ^b^	225.4 ± 16.6 ^b^

**^‡^** Different letter designations (^a, b, c^) within a row represents significant differences (*p* < 0.05) between mean values. For instance, for Arsenic, Yard 1 and Yard 2 with a,b represent no statiscal difference. However, with House and Road, House is significantly different that Road at *p* < 0.05.

As previously mentioned, metals are naturally present in trace amounts within soil matrices with variations depending on geographic area and soil type. Due to this fact, it was important to provide comparison numbers and reference levels for residents. In eighty-four soil samples for the twenty-one residences (four samples per residence), four of seven metals found to be present in SE soil at levels above the normal Franklin County background levels are often associated with the historical use of Pb-based paint and leaded gasoline. This in fact may be the most important finding of this work, and see Figure 4 for an appreciation of the spatial distribution of sampled residences that are in blue. Lead in urban soils comes from the historic use of Pb-based products, including Pb-based paint and Pb-based gasoline. Dried Pb paint often contained 30%–50% Pb by weight. As is shown in Table 1, cadmium (Cd), chromium (Cr), copper (Cu), lead (Pb), molybdenum (Mo), selenium (Se) and zinc (Zn). The metals were also ranked against one another based on soil sampling location. Magnesium was found to be present at above normal levels in the road samples, whereas cadmium, lead and zinc were above normal Franklin County background levels in the three house samples. Paint analyses from older urban homes show high concentrations of multiple metals in exterior paint samples (in mg/kg): Pb 35,000; Zn 31,000; Cd 439; Cu 2,000; and Cr 775 [73,74,75] We found elevated Pb, Cd and Zn, all paint components, at significantly higher levels along the dripline of the homes (*i.e*., house sample). Leaded gas emissions travel more than 100 m from roadways. Therefore, it is possible that the increased levels of soil Pb for the entire neighborhood were due to vehicle emissions from leaded gas. Selenium, thallium and molybdenum were slightly increased in the soils. These trace elements are not associated with Pb-based paint or Pb gas emissions. Atmospheric deposition of coal combustion or other high temperature combustion activities can lead to the elevation of all seven of the metals found in this study (including Mo, Se and Tl). Increased levels of Zn along the house dripline are consistent with the use of Pb-based paint. Although not significant at *p* < 0.05, many soils along the house dripline had enhanced levels of Pb, Cd and Cu, which are all consistent with Pb-based paint use. The enhanced Mg concentrations in the road samples were likely due to dolomitic limestone used for road resurfacing. 

As part of our community-state agency-academic partnership, it was essential that the results be shared with the residents who elected to participate in the soil sampling study. Therefore, an educational, risk communication informational card was developed. This process is complex in nature, because there are different levels of how to report scientific information. If data are included, there must be additional information provided that can provide context and meaning to quantitative numbers. Scientific research is often complex; therefore, possible barriers to communication may arise. It is important to report the facts and the message at a level that can be easily understood by the recipient. It is essential to continue to encourage engagement and to develop trust within the Stambaugh-Elwood community. Materials that are distributed must provide information in an understandable way that will take into account variations in the health literacy of community members.

It is essential to create materials that will not consume the reader or potentially confuse the reader, causing him or her to misinterpret the results. In order to adequately reach an audience, one must consider the quantitative information that will be provided in conjunction with qualitative information that will address potential concerns, interests, vulnerabilities and values. Elliot R. Churchill of the Centers for Disease Control and Prevention suggests a method known as the “single over-riding communications objective.” This method was used in the development of communication materials within the Southern Gateway, because the goal is to educate and inform residents and stakeholders. This method differs from the traditional scientific report method that includes: an Introduction, Materials and Methods section, Results and Discussion. The scientific report method is most applicable in the present study to report preliminary results [76]. 

### 3.3. Citizen Science Risk Communication Activities

**Figure 15:**
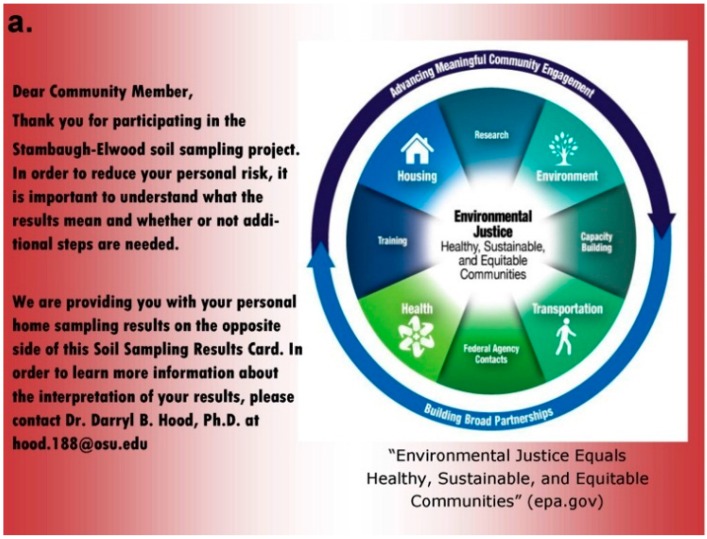

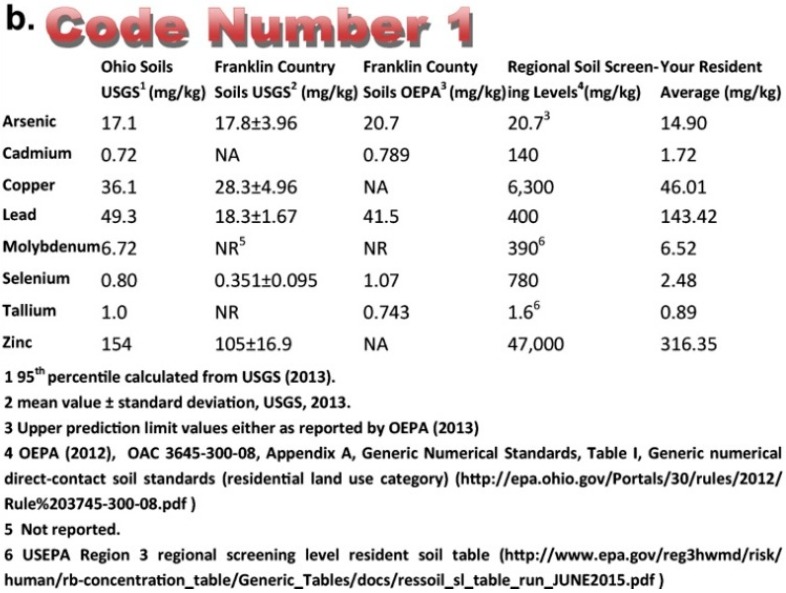
(**a**,**b**) Informational postcard developed and customized for residents of the Stambaugh-Elwood community reporting soil sampling results. (**a**) The front side of the post card; (**b**) the individual residents’ soil sampling result presented consistent with the “single over-riding communications objective concept” developed by Elliot R. Churchill of the Centers for Disease Control and Prevention.

Once all soil samples had been analyzed, results were reported back to the participants in the form of an informational postcard (Figure 15) to answer questions such as: What is the level of lead in my soil? Can I consume vegetables grown in my soil? To do this, residents were invited to a South Side Health Advisory meeting where they were given an informal presentation on the: (1) methodologies used to prepare and analyze their residential soil samples; (2) exposure assessment and risk characterization calculations used to interpret their data (Figure 7, Figure 8, Figure 9, Figure 10, Figure 11, Figure 12, Figure 13 and Figure 14); and (3) an introduction to the format in which their results would be presented. Next, residents were given tailored personalized informational cards (Figure 15a,b) that contained the “raw” confidential data (*i.e.*, milligrams of metal per kilogram of soil, as well as a table that outlined the reference concentrations of metal that their soil sample was analyzed against. The informational card included the following information: individual soil sampling specific to each resident, United States Geological Survey Ohio-specific averages for metals; Franklin County, OH, averages for metals; Ohio-EPA averages for metals; and Ohio-EPA residential soil screening levels (RSLs) of the eight metals. Individual informational postcards conveyed the levels of metals in comparison to these aforementioned reference levels. 

One of the most important aspects of communication efforts at all levels is adequate framing of an issue. According to Taylor, “framing refers to the process by which individuals and groups, identify, interpret, and express social and political concerns.” [77]. Proper framing during communication may positively influence the influx of supporters and community stakeholders that identify with a particular issue. With the release of EJSCREEN by the USEPA, we observed an immediate effect on the individual behavior and collective action of the residents in this community [77]. This type of public participation in scientific research is in general a form of “citizen science”, broadly defined in this study as a partnership between scientists and non-scientists in which authentic data are collected, shared and analyzed [78,79,80]. The present citizen science project was designed to increase the overall health literacy of Stambaugh-Elwood residents; specifically, to increase the science literacy via analysis of residential urban soil samples and to better communicate the potential risk from exposure to environmental contaminants to public health officials via participation in local decision-making venues. Previous research in sociology and science education and has demonstrated the requirement to engage communities in scientific research and that this level of engagement can be successfully facilitated via community-academic partnerships. Members of the Stambaugh-Elwood community, which is located in the midst of industrial smoke-emitting facilities, were motivated to learn more about their potential modes of exposure. These residents were clearly motivated to engage in the process because of being in the proximity of emissions [81]. Just in the new millennium, popular epidemiology was proposed as a community-driven practice by populations experiencing disproportionate contamination and demanded the inclusion of investigations, the gathering of scientific knowledge and the recruiting of scientific professionals [82]. This approach in combination with that specified by “street science” addresses environmental health justice issues that serve to join local knowledge with professional techniques to re-value forms of knowledge that professional science has traditionally excluded [83]. 

The community engagement activity that occurred as a result of this preliminary study will continue to foster a community-state agency-academic partnership within Southern Gateway communities. It is essential that our current and future relationship with these communities be focused on beneficial reciprocal outcomes with a common goal. In addition to providing clear communication materials, it is important to continue to provide communities with assistance and access to informational resources that may enhance knowledge. Community Campus Partnerships for Health, a non-profit organization that promotes health equity and social justice, developed a framework for enhancing university partnerships that consist of nine principles [84]. 

Principles 5 and 7 framed the development of our informational postcard, which ensured that results were conveyed with relevant background information and accessible university and state agency contacts for the purpose of clarification and to provide community members with continued avenues to ask questions or express concerns.

## 4. Conclusions 

Because the results from soil sampling demonstrated that eight metals (As, Cd, Cu, Pb, Mo, Se, Tl, Zn) occurred at statistically-significantly greater levels than natural Franklin County background levels, we will pursue a full hazard identification campaign for this community. Presently, we are in discussions with the Ohio-EPA to partner in the conduct of outdoor particulate air monitoring for the Stambaugh-Elwood community. Although most soil samples were below risk-based residential soil screening levels, the documented disparate health outcomes for Southern Gateway communities warrant such an approach going forward (Figure 1). In consideration of the fact that when using the risk-based methodology, soil metal levels tended to be typical of urban soils and unremarkable, this should not detract from the overall context of our citizen science-GIS findings using MapplerX and those from the USEPA (EJSCREEN). Those findings indicated that the EJ index for the SE community (43,207) is approaching the U.S. 80th percentile in five of seven categories that likely signal an “environmental vulnerability.” 

From a national public health perspective, there are many other communities with characteristics similar to those in SE exhibiting concerns about a myriad of environmental justice issues. As a community becomes more aware of the relationships between potential environmental hazards and potential exposures, it is important to continue to work towards developing effective educational and environmental interventions that improve the health equity of a community. The Environmental Protection Agency’s most recent 2014 Environmental Justice Progress Report developed a framework for including environmental justice concerns in policy making, enforcement and community programming. In May 2015, the USEPA updated this framework to include three environmental justice priorities that the agency aims to focus on in the next five years. The USEPA has committed to reducing environmental health disparities, developing community relationships and identifying progress in burdened areas. In order to provide quality hazard identification activities leading to risk assessments, various factors should be taken into account, such as population susceptibility, socioeconomics and cumulative exposure [54,85,86]. It will be essential to continue to partner with community members in identifying resources towards the implementation of effective environmental-based interventions that better inform public health policy. Community-led coalitions that collaborate and partner with academic teams and state agencies have the potential to increase health literacy, reduce environmental health disparities and increase movement towards the goal of health equity.
